# Burst of Corneal Dendritic Cells during Trastuzumab and Paclitaxel Treatment

**DOI:** 10.3390/diagnostics11050838

**Published:** 2021-05-07

**Authors:** Katharina A. Sterenczak, Nadine Stache, Sebastian Bohn, Stephan Allgeier, Bernd Köhler, Andreas Bartschat, Christian George, Rudolf F. Guthoff, Oliver Stachs, Angrit Stachs

**Affiliations:** 1 Department of Ophthalmology, Rostock University Medical Center, 18057 Rostock, Germany; nadine.stache@uni-rostock.de (N.S.); sebastian.bohn@uni-rostock.de (S.B.); rudolf.guthoff@med.uni-rostock.de (R.F.G.); oliver.stachs@uni-rostock.de (O.S.); 2 Department of Obstetrics and Gynecology, University of Rostock, 18059 Rostock, Germany; christian.george@kliniksued-rostock.de (C.G.); angrit.stachs@uni-rostock.de (A.S.); 3 Department Life, Light & Matter, University of Rostock, 18059 Rostock, Germany; 4 Institute for Automation and Applied Informatics, Karlsruhe Institute of Technology, 76021 Karlsruhe, Germany; stephan.allgeier@kit.edu (S.A.); bernd.koehler@kit.edu (B.K.); andreas.bartschat@kit.edu (A.B.)

**Keywords:** breast cancer therapy, neuropathy, ocular complications, *in vivo* large-scale confocal laser scanning microscopy, dendritic cells

## Abstract

During breast cancer therapy, paclitaxel and trastuzumab are both associated with adverse effects such as chemotherapy-induced peripheral neuropathy and other systemic side effects including ocular complications. Corneal nerves are considered part of the peripheral nervous system and can be imaged non-invasively by confocal laser scanning microscopy (CLSM) on the cellular level. Thus, *in vivo* CLSM imaging of structures of the corneal subbasal nerve plexus (SNP) such as sensory nerves or dendritic cells (DCs) can be a powerful tool for the assessment of corneal complications during cancer treatment. During the present study, the SNP of a breast cancer patient was analyzed over time by using large-scale *in vivo* CLSM in the course of paclitaxel and trastuzumab therapy. The same corneal regions could be re-identified over time. While the subbasal nerve morphology did not alter significantly, a change in dendritic cell density and an additional local burst within the first 11 weeks of therapy was detected, indicating treatment-mediated corneal inflammatory processes. Ocular structures such as nerves and dendritic cells could represent useful biomarkers for the assessment of ocular adverse effects during cancer therapy and their management, leading to a better visual prognosis.

**Background**: Cancer is the leading cause of death in the developed world, and besides the traditional non-surgical treatments, including radiation and chemotherapeutic drugs, the current trend is focused on using targeted biological therapies eliminating cancer cells only or interfering with tumor growth and progression by specific molecules or antibodies [1,2]. Although biological therapy is less toxic to healthy cells compared to chemotherapy, biological treatments can also lead to numerous systemic side effects [1,2].

In human epidermal growth factor receptor (HER)-2-positive breast cancer, the standard systemic treatment consists of the combination of paclitaxel-based chemotherapy and an anti-HER-2-antibody treatment using trastuzumab. One of the potentially dose-limiting side effects of paclitaxel treatment is chemotherapy-induced peripheral neuropathy (CIPN). The predominantly sensory neuropathy can affect large and small nerve fibers by damaging large-diameter sensory myelinated (Aβ) fibers or dorsal root ganglia (DRG) cells [3]. Although improvement of nerve function after canceling cytostatic therapy is possible, sometimes CIPN causes permanent impairment of quality of life. The gold standard in the diagnosis of CIPN is nerve conduction studies with regard to the sural nerve. For early/preclinical detection of CIPN, no validated biomarker exists yet. Ferdousi et al. reported small fiber neuropathy of corneal nerve plexus detected by *in vivo* confocal laser scanning microscopy (CLSM) in patients with upper gastrointestinal cancer and treatment with platinum-based chemotherapy [4]. In a very recent study by Chiang et al. [5], *in vivo* CLSM revealed a significant reduction in corneal nerve fibers in patients with paclitaxel-induced neuropathy. Moreover, corneal changes detected by *in vivo* CLSM were also found in breast cancer patients treated with trastuzumab [6,7,8].

The cornea is considered to be the most densely innervated tissue in the human body, and corneal nerves, which arise from the ophthalmic branch of the trigeminal nerve, are considered part of the peripheral nervous system [9]. Native corneal nerves can be imaged non-invasively by *in vivo* CLSM. Moreover, this method offers the ability to examine the same corneal region repeatedly over time without causing tissue damage. *In vivo* CLSM generates surface-parallel images of the native cornea with cellular resolution, and it can be used to image dendritic cells (DCs) as well as nerves from the subbasal nerve plexus (SNP) with high contrast [10]. Moreover, several studies have shown that SNP changes are not characteristic of one specific corneal pathology but rather reflect non-specific pathological processes that are present in many corneal, ocular, or systemic diseases [11,12,13] or arise as a result of a therapy regime, such as that used to treat multiple myeloma [14] or breast cancer [6,7,8]. Thus, time-lapsed *in vivo* CLSM-based cell imaging of biomarkers such as corneal sensory nerves or DCs could be a promising tool for the early detection of corneal changes in patients. Consequently, corneal *in vivo* CLSM could be used as a surrogate or even prognostic marker for CIPN or ocular adverse events in association with antibody-drug administration.

During conventional *in vivo* CLSM, a single image of 0.16 mm^2^ covers approximately 0.2% of the average corneal surface, which is insufficient for reliable morphometric assessment of the complete SNP [11]. Moreover, the technique is very dependent on the skills of the operator, and the imaging of the same topographical location in repeated examinations is randomly or nearly impossible. Within this study, *in vivo* large-area CLSM imaging was applied, generating mosaic images covering between 3 and 4 mm^2^ of the SNP [15,16]. This report represents the first longitudinal evaluation of the SNP of a HER-2 positive breast cancer patient during paclitaxel and trastuzumab therapy by *in vivo* large-area CLSM.

**Patient history:** A 52-year-old woman with HER-2-positive breast cancer has received adjuvant cytotoxic chemotherapy in combination with Anti-HER-2 antibody (trastuzumab) therapy. The patient has a body mass index of 33.9 (adipositas grade I) and no further significant history of alcohol abuse, diabetes, diseases of the nervous system, systemic conditions, or further medical features. The regime of the chemotherapy was paclitaxel 80 mg/m^2^ weekly and trastuzumab, starting with a loading dose of 8 mg/kg followed by 6 mg/kg every 3 weeks. Seven courses of paclitaxel and three courses of trastuzumab were administered. Paclitaxel therapy was stopped because the patient developed relevant CIPN. Antibody treatment was continued for a total of one year. The cumulative dose of paclitaxel was 1097.6 mg and of trastuzumab 1820 mg. For surveillance of a possible occurrence of polyneuropathy under paclitaxel treatment, nerve function sensitivity was evaluated regularly by the neuropathy symptom score (NSS) and the neuropathy deficit score (NDS). The assessment included specific questions regarding neurological symptoms (sensory, motor) with particular attention to neuropathic pain and paraesthesia occurrence, patella and Achilles tendon reflex, sense of vibration and thermoregulatory disturbance, and sensation in the feet. While the NDS score remained low, the NSS score ranged from mild at the onset of therapy to severe at 11 weeks after the start of therapy. The patient was regularly seen in our outpatient eye-clinic and underwent a complete ophthalmologic examination, i.e., determination of visual acuity, intraocular pressure, corneal esthesiometry (Cochet-Bonnet), and slit-lamp examination with fundoscopy showing no clinically relevant findings.

**Large area *in vivo* CLSM of the patient’s cornea:** The patient underwent *in vivo* large-area CLSM at three separate sessions, with subsequent morphometric assessment of the subbasal nerve plexus (SNP). The follow-up sessions took place 6 weeks (47 days) and 11 weeks (78 days) after the baseline date. The imaging system combines a Heidelberg Retina Tomograph 3 (HRT 3, Heidelberg Engineering GmbH, Heidelberg, Germany) with the experimental Rostock Cornea Module 2.0 (modified RCM, Heidelberg Engineering GmbH, Heidelberg, Germany) and the EyeGuidance system [15,17,18]. During image acquisition, the EyeGuidance system presents a moving fixation target to the contralateral eye, inducing smooth pursuit eye movements (of both eyes) along a predefined spiral path. By using the recorded image data, the mosaic images are generated in a subsequent process. An in-depth description and discussion of the image acquisition and processing procedure is available in [15]. The assignment of identical areas in the mosaic images taken at different sessions was done by comparing the present ridge-like deformations within the SNP, which were described by Kobayashi [19] and termed K-structures. These structures strongly align with the anterior corneal mosaic (ACM) which was first described by Bron in 1968 [20,21]. The ACM can be observed by slit-lamp examination after instillation of fluorescin into the conjunctival sac and a massage of the cornea on the surface of the corneal epithelium through the lids [20]. This ACM pattern disappears after a short period of time and can be re-induced again after pressure on the cornea [20]. *In vivo* CLSM also induces pressure on the corneal surface via the contact element (TomoCap; Heidelberg Engineering, Heidelberg, Germany), leading to the formation of the K-structures at the level of the SNP. The following Figure 1 and Figure 2 show the SNP of our patient during paclitaxel and trastuzumab treatment by *in vivo* large-area CLSM.

**Increase in DC density and a burst at 11 weeks of therapy:** While the nerve morphology remained stable during the tracked weeks of therapy, the DC density increased over time and showed a regional burst at 11 weeks (Figure 2). In order to compare the regional differences of DC densities within the same mosaic image after 11 weeks of treatment, the amount of DCs within the white insets (size 500 µm × 500 µm) in Figure 1 was analyzed manually in triplicate. The upper inset, which was located within the region presenting a higher number of DCs, showed a mean of 244 DCs, whereas the lower inset showed a mean of 52 DCs, being 4.6 times smaller than the density within the upper inset. In order to compare DC density over time and within identical areas, a manual quantification of DCs within the red marked areas (Figure 2) was performed using ImageJ and the CellCounter plugin. The number of DCs was analyzed in triplicate, showing medians of 71 DCs at baseline, 171 DCs after 6 weeks, and 457 DCs at 11 weeks of therapy. In summary, the number of DCs increased by about 2.5 times within the first 6 weeks and more than sixfold after 11 weeks of treatment, indicating ongoing inflammatory processes during cancer therapy of the patient.

**Therapy-induced changes in the patient’s SNP:** The *in vivo* large-area CLSM applied herein allowed the monitoring of the patient’s SNP over time. We were able to assign identical areas and could detect a regional burst in DC density increasing with time, indicating local inflammatory processes. Corneal DCs are professional antigen-presenting cells, which play a significant role in the innate and adaptive immune system during corneal homeostasis and wound healing. The morphology and density of DCs change upon stimulation, resulting in maturation and increase in cell size as well as formation and lengthening of dendrites. In an *in vivo* CLSM study by Mastropasqua et al., the density, distribution, and morphology of DCs were analyzed in normal subjects, photorefractive keratectomy (PRK) patients and patients affected by immune-mediated corneal inflammation [22]. DC densities were significantly higher at the limbus compared to the central cornea in each group. Moreover, the DC densities were higher in inflamed eyes at both locations, the central cornea, and the limbus when compared with the normal and the PRK group [22]. The authors described that inflammation of wound healing, for example, after PRK, is likely different to inflammation of infection or immune-mediated reactions as DCs presence and density of PRK eyes was similar to normal eyes as to inflamed eyes [22]. The DC population by eyes affected by immune-mediated inflammation presented major differences compared to the control group. Besides higher densities at the limbus and central cornea, the morphologic features differed as well. The DCs of inflamed eyes showed larger cell size and reflectivity, more dendritic processes, a frequent “cluster-distribution” and possible association with coexisting globular cells compared to control eyes [22]. In the present study, the scans were performed at the central cornea of the patient. The detected DC morphology and increasing density during therapy with a regional burst implies ongoing inflammation or immune-mediated reactions caused by the paclitaxel and/or trastuzumab treatment.

Trastuzumab binds specifically to HER2-positive cancer cells and down-regulates their downstream signaling cascades, leading to inhibition of proliferation and survival [23]. Although antibody drug-conjugates (ADCs) such as trastuzumab were designed to enhance tissue specificity, the presence of similar targets in healthy tissues results in a variety of drug-related toxicities including ocular toxicities [24]. Specifically, HER2 is a member of the human epidermal growth factor receptor (EGFR) family, and HER1 (EGFR), HER2 (ErbB2), and HER3 (ErbB3) have been detected in corneal, limbal, and conjunctival epithelium [25]. Moreover, EGFR signaling has been found to be necessary and sufficient for corneal epithelial migration, proliferation, and differentiation, and examination of different EGFR ligands indicated that epidermal growth factor is the primary mediator of corneal epithelial homeostasis [26]. Considering this pivotal role of EGFR and its ligands on the ocular surface, anti-cancer treatments targeting EGFR likely result in corneal complications. The cytotoxicity is probably related to intracellular accumulation of the active metabolite in normal corneal epithelial cells [8,24]. Trastuzumab-mediated ocular adverse effects include a variety of symptoms such as conjunctivitis, dry eye, increased lacrimation, and blurred vision. [24]. In some recent case reports, progressive corneal ulcerative damage [27], corneal lesions [7], and cystoid lesions in deep corneal epithelium [6] due to treatment with trastuzumab were reported. Most of these adverse effects were described to be stationary and reversible and did not require ocular treatment or cessation of systemic treatment [8].

Notably, in a cross-sectional study regarding corneal features in trastuzumab emtansine treatment by Deklerck et al. [8], hyperreflective lesions likely representing necrotic cells at the level of wing cells and an increased presence of dendritic cells at the level of the SNP were described. According to [8], the increased presence of dendritic cells supports the hypothesis of ADC-associated corneal toxicity with a low-grade local inflammatory response. The results of [8,22,28] support the findings presented here regarding the distribution and morphology of DCs in the patient’s SNP, indicating treatment-mediated local inflammatory response in the cornea. The reasons why these local clusters of DCs occur and why some regions seem to be more attractive than others, however, remain open for discussion within the community. In the future, in-depth longitudinal CLSM studies involving a prospective cohort, where patients are followed prior to, during, and after chemotherapy treatment are required. This will help to translate the gained data and to better understand the nature of these findings and the impact of trastuzumab or paclitaxel.

**Conclusions:** The large-scale *in vivo* CLSM technique used herein enabled the longitudinal detection of the SNP of a patient in the course of the treatment regimen. While the subbasal nerves did not alter significantly, we could detect a change in DC density within the first 11 weeks of treatment. The detected increase in DC density, as well as the local burst, implied an inflammatory response likely caused by trastuzumab treatment. As paclitaxel and trastuzumab were associated with ocular adverse events, baseline and regular follow-up screenings would be of great clinical benefit, and the ideal management of these therapy-induced ocular complications would require a close partnership between oncologists and ophthalmologists. The *in vivo* large-area CLSM applied herein could be used for both the identification of therapy-mediated adverse effects and the longitudinal monitoring of disease progression. Ocular structures such as subbasal nerves and dendritic cells could be of great value as potential biomarkers for the assessment of the severity of adverse effects and the outcome for the patients. Accordingly, patients who start biological treatment should be screened by ophthalmologists on a regular basis. The early detection of ocular side effects during cancer therapy may lead to a better visual prognosis.

## Figures and Tables

**Figure 1:**
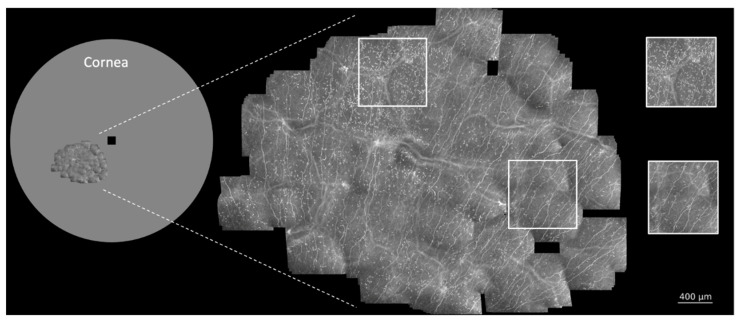
*In vivo* large-area confocal laser scanning microscopy (CLSM) of the subbasal nerve plexus (SNP) of a human epidermal growth factor receptor (HER)-2 positive breast cancer patient during paclitaxel and trastuzumab therapy. Left side: Human cornea, size and location of the performed *in vivo* large-area CLSM scan and comparison to conventional CLSM image size (black square). Right side: The patient’s SNP 11 weeks after the start of therapy. Local differences in dendritic cell (DC) density could be observed. White insets: regional differences in DC density in different areas of the same mosaic image.

**Figure 2:**
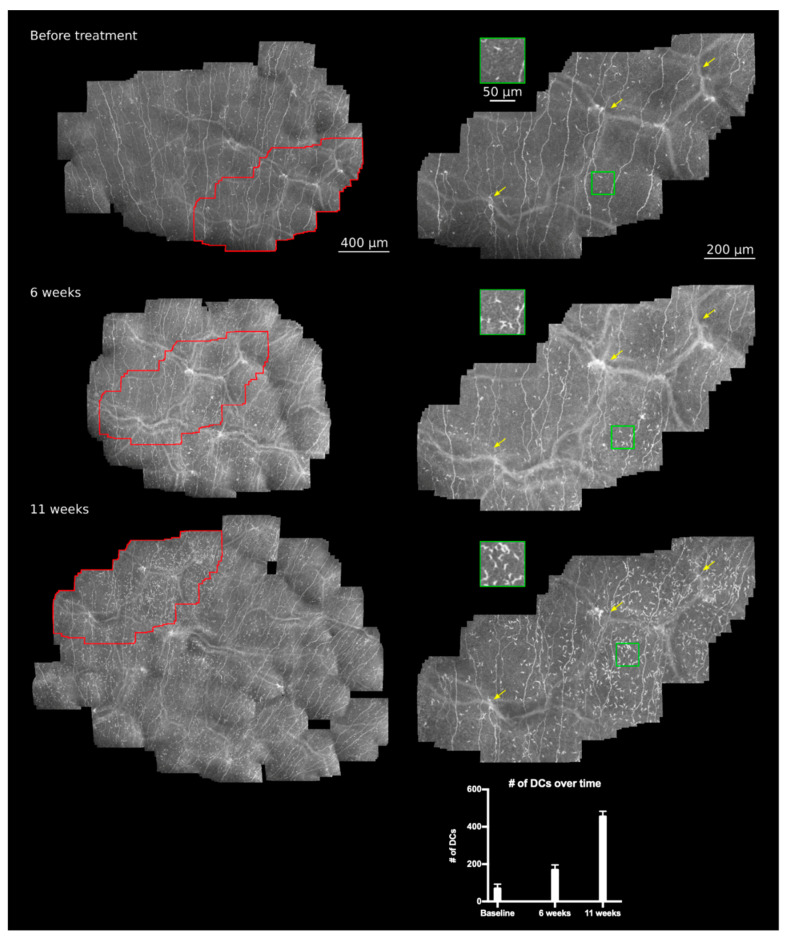
Longitudinal *in vivo* large-area CLSM scans of the SNP of a HER-2 positive breast cancer patient during paclitaxel and trastuzumab therapy. From top to bottom: The patient’s SNP before treatment, at 6 weeks, and at 11 weeks of treatment. Identical areas within all reconstructed images (left side: red areas, right side: enlarged view of red marked areas) could be assigned by characteristic recurring patterns (yellow arrows). During the therapy regimen, the DC density increased and showed a regional burst at 11 weeks after the start of therapy. The bar plot shows the manual count of DC density over time within identical areas. Nerve fiber density and morphology remained stable except for minor fluctuations. Red area: identical area; green area and inset: DC density.

## Data Availability

The data presented in this study are available on request from the corresponding author.
